# Retroperitoneal kidney transplantation with liver and native kidney mobilization: a safe technique for pediatric recipients

**DOI:** 10.1007/s12519-022-00658-7

**Published:** 2022-12-06

**Authors:** Juliano Riella, Raphealla Ferreira, Marina M. Tabbara, Phillipe Abreu, Lucas Ernani, Marissa Defreitas, Jayanthi Chandar, Jeffrey J. Gaynor, Javier González, Gaetano Ciancio

**Affiliations:** 1 Department of Surgery, University of Miami Miller School of Medicine, Jackson Memorial Hospital, Miami, FL USA; 2 Department of Urology, University of Miami Miller School of Medicine, Jackson Memorial Hospital, Miami, FL USA; 3 Department of Pediatrics, Division of Nephrology, University of Miami Miller School of Medicine, Jackson Memorial Hospital, Miami, FL USA; 4 Miami Transplant Institute, University of Miami Miller School of Medicine, Jackson Memorial Hospital, 1801 NW 9Th Ave, 7Th Floor, Miami, FL 33136 USA; 5 Servicio de Urología, Unidad de Trasplante Renal, Hospital General Universitario Gregorio Marañón, Madrid, Spain

**Keywords:** Extraperitoneal approach, Liver and kidney mobilization, Pediatric kidney surgical technique, Transplantation

## Abstract

**Background:**

Pediatric kidney transplant (KT) using larger, deceased or living donor adult kidneys can be challenging in the pediatric population due to limited space in the retroperitoneum. Liver and native kidney (L/NK) mobilization techniques can be used in smaller and younger transplant recipients to aid in retroperitoneal placement of the renal allograft. Here, we compare the clinical outcomes of pediatric retroperitoneal KT with and without L/NK mobilization.

**Methods:**

We retrospectively analyzed pediatric renal transplant recipients treated between January 2015 and May 2021. Donor and recipient demographics, intraoperative data, and recipient outcomes were included. Recipients were divided into two groups according to the surgical technique utilized: with L/NK mobilization (Group 1) and without L/NK mobilization (Group 2). Baseline variables were described using frequency distributions for categorical variables and means and standard errors for continuous variables. Tests of association with the likelihood of using L/NK mobilization were performed using standard *χ*^2^ tests, *t* tests, and the log-rank test.

**Results:**

Forty-six pediatric recipients were evaluated and categorized into Group 1 (*n* = 26) and Group 2 (*n* = 20). Recipients in Group 1 were younger (6.7 ± 0.8 years vs. 15. 3 ± 0.7, *P* < 0.001), shorter (109.5 ± 3.7 vs. 154.2 ± 3.8 cm, *P* < 0.001) and weighed less (21.4 ± 2.0 vs*.* 48.6 ± 3.4 kg, *P* < 0.001) than those in Group 2. Other baseline characteristics did not differ between Groups 1 and 2. One urologic complication was encountered in Group 2; no vascular or surgical complications were observed in either group. Additionally, no stents or drains were used in any of the patients. There were no cases of delayed graft function or graft primary nonfunction. The median follow-up of the study was 24.6 months post-transplant. Two patients developed death-censored graft failure (both in Group 2, *P* = 0.22), and there was one death with a functioning graft (in Group 2, *P* = 0.21).

**Conclusions:**

Retroperitoneal liver/kidney mobilization is a feasible and safe technique that facilitates implantation of adult kidney allografts into pediatric transplant recipients with no increased risk of developing post-operative complications, graft loss, or mortality.

## Introduction

Kidney transplantation (KT) remains the gold-standard treatment for pediatric patients with end-stage kidney disease (ESKD), as it is associated with fewer cognitive and learning impairments in addition to improved patient survival when compared to remaining on dialysis [1–3]. However, the waiting list for a deceased donor (DD) KT in pediatric patients is very long due to the need for and relative scarcity of an age- and/or size-matched graft. The utilization of adult-sized kidneys in this population provides a valid yet technically challenging alternative [4]. Placing an adult-sized kidney into a small child is classically performed via a midline laparotomy with intraperitoneal implantation due to space limitations [5]. This approach is technically difficult due to the high prevalence of prior abdominal operations in this pediatric population [6], and it carries a higher risk of developing abdominal compartment syndrome, early vascular compromise of the allograft, and other nonrenovascular complications [7–9].

In efforts to mitigate the risk of bowel obstruction and iatrogenic bowel injury [10, 11], retroperitoneal KT has been described as a feasible alternative. However, this technique has been historically associated with the need for a native nephrectomy in efforts to create space for renal allograft placement in pediatric patients. Alameddine et al. [11] presented a retroperitoneal approach with mobilization of the liver and native kidney, avoiding native nephrectomy and its possible complications and creating retroperitoneal space to accommodate an adult kidney graft.

In this study, we retrospectively evaluated pediatric renal transplant recipients who underwent retroperitoneal KT of adult donor allografts and either received or did not receive liver/native kidney (L/NK) mobilization (based upon need). The clinical outcomes of the two approaches were compared and are reported here.

## Methods

This retrospective study aimed to evaluate the clinical outcomes of pediatric retroperitoneal KT with and without L/NK mobilization. All pediatric recipients (age of 18 years or less) who underwent KT between January 2015 and May 2021 were included. This study was approved by the Institutional Review Board at the University of Miami and follows the Helsinki Declaration (as revised in 2013). Written informed consent was obtained from all subjects’ parents or a legal surrogate. The last follow-up date for the study was June 3, 2022.

### Pretransplant workup

Recipients were evaluated by a pediatric transplant nephrologist and transplant surgeon, both of which determined the surgical risk assessment and technical feasibility of performing the transplant surgery. Pediatric recipients underwent extensive laboratory workup, including complete blood count, comprehensive metabolic panel, electrolytes, serologies, echocardiogram, EKG, chest X-ray, and abdominal ultrasound, and ultimately informed consent from the parents.

### Study groups

Patients were categorized into two groups according to the surgical technique performed. Group 1 was defined as recipients who underwent KT with retroperitoneal L/NK mobilization. The mobilization technique included the creation of a retroperitoneal space posterior to the right kidney and liver to accommodate the adult allograft. Group 2 was defined as recipients who underwent retroperitoneal KT without L/NK mobilization. Surgical technique (group) was determined intraoperatively depending on the available retroperitoneal space and body habitus of the patient to accommodate the size of the adult allograft; thus, no patient randomization to standard technique vs. L/NK mobilization prior to surgery or intraoperatively was performed.

### Surgical technique

**Fig. 1:**
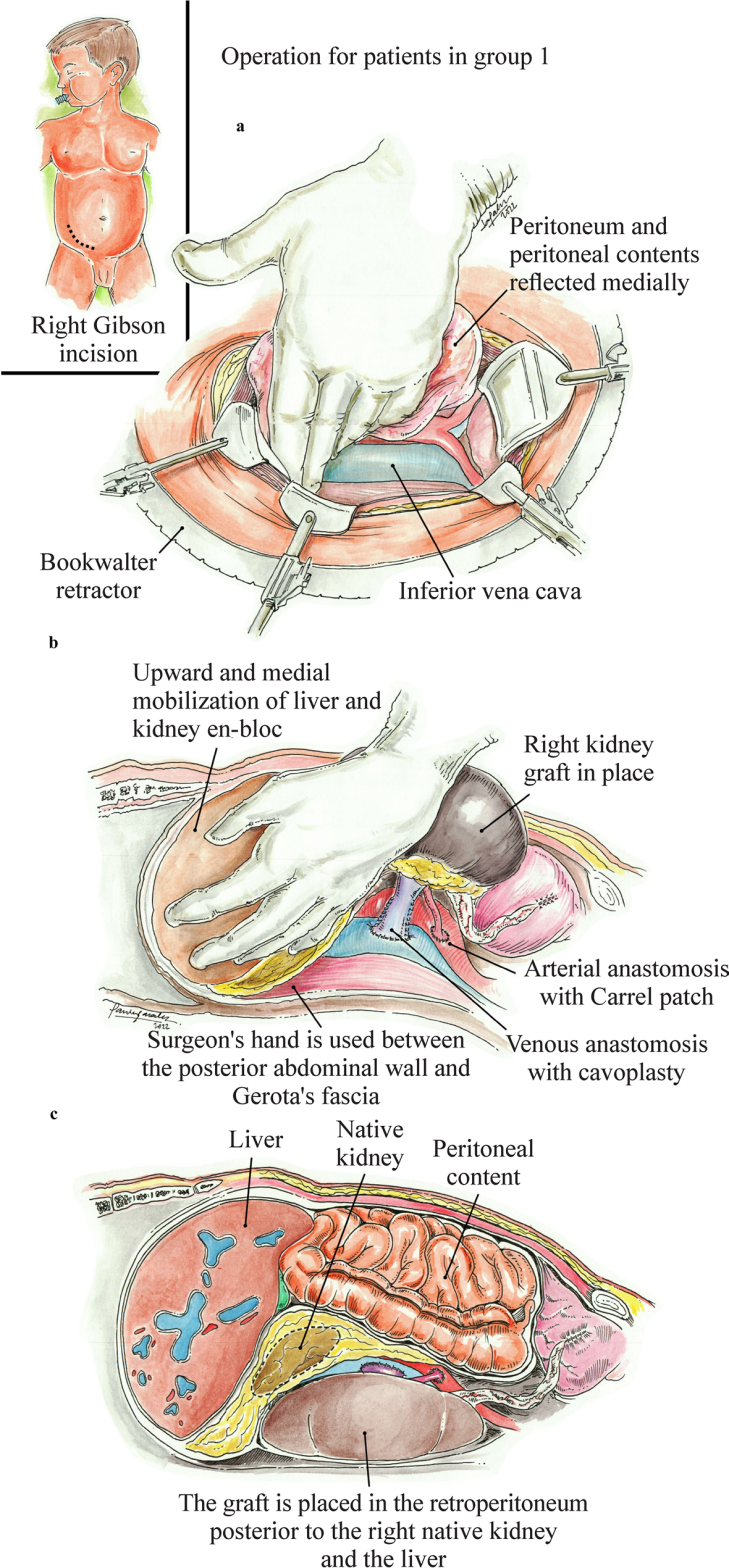
Surgical procedure for Group 1 pediatric kidney recipients. **a** Gibson incision, reflecting the peritoneal content medially exposing the inferior vena cava and the right common iliac artery; **b** Mobilization of the liver and kidney en-bloc to create a new retroperitoneal space; **c** Placement of an adult kidney allograft in the new retroperitoneal space

The surgical technique for retroperitoneal KT with L/NK mobilization (Group 1) has been previously described in detail by Alameddine et al. [11]. A Gibson incision is made on the right lower quadrant that facilitates access to the inferior vena cava (IVC). The abdominal wall is opened in layers until the peritoneum is found, which is then reflected medially. Blunt dissection is carried out cephalad posterior to the right renal fossa. This maneuver exposes the aorta, IVC and right iliac vessels. A Bookwalter retractor is used to facilitate exposition. The surgeon’s hand can be used between the posterior abdominal wall and Gerota’s fascia, and upward, medial mobilization of the liver en bloc with the native kidney is performed. This creates enough space for the graft and exposes the vascular anatomy, which will be suitable for anastomosis (Fig. 1).

The renal allograft is prepared on the back table. The perinephric fat is trimmed, the renal artery and any accessory artery are identified and preserved, and an aortic Carrel patch is used routinely with the deceased donor kidney. In the case of multiple renal arteries, vascular reconstruction with the intent of achieving a single orifice is performed with polypropylene 8–0. The renal vein is isolated, and branches are ligated. If a deceased right kidney allograft is used, cavoplasty is performed using 6–0 prolene sutures on each side of the cava to create a longer vein. The ureter is identified, and minimal dissection is performed. Then, in the recipient, vascular control is obtained with non-traumatic vascular clamps. The allograft is then brought to the field; the first anastomosis is the between the donor renal artery to the recipient aorta or right common iliac artery, with the decision of which target being based on vessel size, position and length of the retrieved donor renal artery, aiming to avoid kinking and an easy anastomosis with proper size match using continuous 7–0 polypropylene sutures. Then, anastomosis between the retrieved donor renal vein and the recipient IVC is performed using running 6–0 polypropylene sutures. Reperfusion is obtained with attention to the patient’s mean arterial pressure, ideally kept > 90 mmHg at the time of blood flow restoration. The renal allograft is then positioned in the retroperitoneum posterior to the right native kidney and liver. The ureter is then anastomosed to the recipient bladder using the Miami Transplant Institute (MTI) ureteral technique [12].

**Fig. 2:**
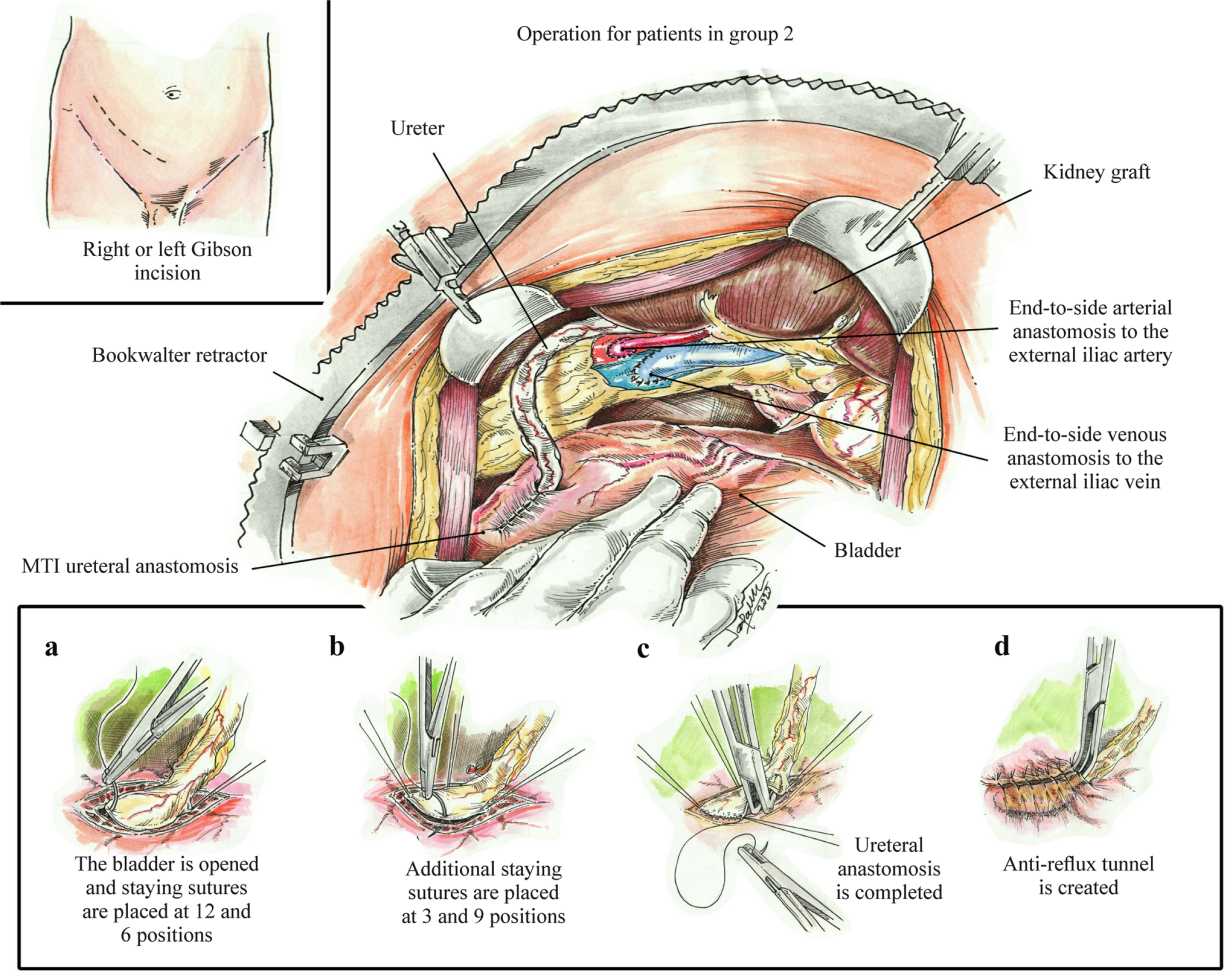
Surgical procedure for Group 2 pediatric kidney transplant recipients. Renal allograft transplantation in the retroperitoneal space without liver and kidney mobilization. Including a description of the Miami Transplant Institute (MTI) ureteral anastomosis

The surgical technique for patients undergoing KT without L/NK mobilization (Group 2) is described as a standard retroperitoneal technique with a Gibson incision in the right or left lower quadrant. The abdominal wall muscles are then incised, and the peritoneum is reflected medially, with blunt dissection of the retroperitoneum creating enough space to accommodate the allograft and exposing the iliac vessels. A Bookwalter^®^ retractor is then placed, and the right external iliac artery and vein are isolated and dissected as minimally as possible to accommodate the non-traumatic vascular clamps and allow space for vascular anastomosis. The graft is then brought to the field, and arterial anastomosis is performed end-to-side between the renal artery and right external iliac artery (EIA) using 6–0 polypropylene sutures in a running fashion. The renal vein is then anastomosed to the recipient right external iliac vein (EIV) using 5–0 continuous polypropylene suture. After reperfusion, the ureter is trimmed and anastomosed to the bladder via MTI ureteral anastomosis (Fig. 2) [12].

### Living donor technique

In cases where an allograft was obtained from a living donor (LD), donor nephrectomy was performed via a hand-assisted laparoscopic approach with lateral decubitus positioning of the donor. The recipient surgery is performed by obtaining adequate exposure of the retroperitoneum and iliac vessels as described above. After placing the graft on a basin with ice, the staple lines are removed from the renal artery and vein, and the graft is flushed with cold histidine-tryptophan-ketoglutarate until the effluent is clear. Once the renal vessels are prepared, the kidney is brought to the field, and the implantation proceeds as previously discussed.

Multiple renal arteries (MRA) were encountered in nine allografts; of those, four were DD grafts and five were LD grafts. All four DD allografts (2 in Group 1 and 2 in Group 2) had a main renal (RA) and one or more accessory arteries that were reconstructed into a single aortic Carell patch. Of the 5 living donor allografts with MRA, several techniques were performed to create a single ostium for implantation in efforts to decrease warm ischemia time [13]. In Group 1 (*n* = 2), one kidney graft had a very short upper pole RA, so the ipsilateral inferior epigastric artery of the recipient was used as an interposition graft, which was anastomosed to the main RA in an end-to-side fashion. The second kidney had two RA that were too far apart to perform a single anastomosis; therefore, they were anastomosed separately to the right common iliac artery (CIA) and right EIA. In Group 2 (*n* = 3), one kidney graft had two main RA that were conjoined into a single ostium. The second kidney graft had a short accessory RA that was anastomosed end-to-side to the main RA. Last, the third kidney graft had 3 RA–the lower pole RA was anastomosed end-to-side to the main RA, and an upper pole RA was anastomosed end-to-side to a branch of the main RA inside the hilum. All vascular reconstructions were performed using 8–0 polypropylene sutures.

No ureteral stents or Jackson-Pratt drains were utilized in any of the cases [12, 14]. All patients received a nasogastric tube during the procedure that was removed after extubation.

### Hypothermic machine perfusion

For the cases in which DD renal allografts were used, once received at our center from the allocation, the kidney was connected to the LifePort^®^ renal preservation machine (Organ Recovery Systems, Itasca, IL, USA) and stored in hypothermia (2–4 °C) using kidney preservation solution (KPS-1) [15].

### Immunosuppression

All children received induction immunosuppression in the form of antithymocyte globulin 1 mg/kg (3 doses), basiliximab 10 mg (2 doses) and methylprednisolone starting at 10 mg/kg (maximum 500 mg/dose) [16]. Methylprednisolone was tapered by 2 mg/kg daily and discontinued by post-operative days 5–7, when a therapeutic level of tacrolimus was obtained (target level 6–8 ng/mL). Most patients received a steroid-free maintenance regimen that included tacrolimus and mycophenolate mofetil. Oral tacrolimus was introduced when serum creatinine was < 3 mg/dL in children older than 6 years of age and < 2 mg/dL in those who were younger. Oral mycophenolate mofetil was introduced on the second post-operative day at a dose of 600 mg/m^2^ twice daily. Dose adjustment was performed according to white blood cell count and gastrointestinal tolerance.

### Post-transplant workup

All patients underwent a renal ultrasound 24–48 hours post-transplantation to evaluate the development of any fluid collections and to assess renal artery and renal vein flow. Depending upon clinical suspicion, a percutaneous allograft biopsy was performed by Interventional Radiology and reviewed by an experienced pathologist and reported based on the 2019 Banff revised classification [17].

### Baseline variables and clinical outcomes

Baseline variables that were studied included donor and recipient demographics, clinical characteristics of the underlying disease of recipients, donor kidney anatomic pathological evaluation, and pre- and intraoperative data, including history of surgical intervention pretransplant, history of bladder augmentation, type of arterial and venous anastomosis, warm ischemia time (WIT), cold ischemia time (CIT), and estimated blood loss (EBL). Serum creatinine was obtained at 6 and 12 months. The estimated glomerular filtration rate (eGFR) was calculated using Schwartz’s original formula, where the constant of proportionality (k) was adjusted for the child’s age and sex [18]. Acute rejection was diagnosed after a clinically indicated renal allograft biopsy was evaluated by an experienced pathologist. Delayed graft function (DGF) was defined as the need for dialysis during the first 7 days post-transplant. Primary graft nonfunction (PNF) was determined as ongoing DGF that required ongoing dialysis dependency 3 months post-transplant. Complications (vascular or urologic) that developed within 30 days of kidney transplant were included and classified based on previously published Dindo-Clavien classification [19]. Recipient outcomes, including the development of post-operative complications and graft and patient survival, were collected and analyzed.

### Statistical analysis

Baseline variables were described using frequency distributions for categorical variables and means and standard errors for continuous variables (geometric means and corresponding standard errors were used for skewed distributions). Medians and ranges of continuous variables were also provided. Tests of association with the likelihood of using L/NK mobilization (yes/no) were performed using Pearson chi-squared tests for categorical variables, standard *t* tests for continuous variables, and log-rank tests for time-to-event variables. *T* tests comparing geometric means were performed using natural logarithmic transformed values. *P* values < 0.05 were considered to be statistically significant.

## Results

### Combined cohort

Distributions of selected of recipient, donor, perioperative, and surgical technique variables are described in Table 1. 58.7% of the recipients were male (27/46), with a mean recipient age of 10.4 ± 0.8 years. Most patients were receiving renal replacement therapy (RRT) (80.4%, 37/46), with a mean time on RRT of 1.8 ± 0.2 years (median 1.5, range: 0.0–6.0 years). The mean recipient height and weight were 128.9 ± 4.2 cm (median 126.5, range: 82.0–180.0 cm) and 33.2 ± 2.7 kg (median 26.5, range 11.0–85.0 kg), respectively. The percentage of patients classified as having intrinsic renal pathology as the etiology of ESKD was 50% (23/46), with 4.3% (2/46) patients diagnosed with recurrent ESKD.

**Table 1:** Distributions of selected baseline (recipient, donor, perioperative, and surgical technique) variables (*N* = 46)

Baseline variables	Percentage with characteristic for categorical variables (mean ± SE for continuous variables and geometric mean ± SE for skewed continuous variables); (median and range are included for continuous variables)
Recipients	
Recipient male	58.7% (27/46)
Recipient age (y)	10.4 ± 0.8 (*N* = 46) (median = 10.0, range: 2–19)
Received RRT	80.4% (37/46)
Years on RRT (0 if none)	1.8 ± 0.2 (*N* = 46) (median = 1.5, range: 0.0–6.0)
Recipient height (cm)	128.9 ± 4.2 (*N* = 46) (median = 126.5, range: 82.0–180.0)
Recipient weight (kg)	33.2 + 2.7 (*N* = 46) (median = 26.5, range: 11.0–85.0)
ESRD diagnosis	
Pre-renal	4.3% (2/46)
Renal	50.0% (23/46)
Post-renal	17.4% (8/46)
Combined	21.7% (10/46)
Unknown	6.5% (3/46)
Recurrent ESRD	4.3% (2/46)
Donors	
Kidney length (cm)	11.1 ± 0.2 (*N* = 37) (median = 11.0, range: 9.0–14.0)
Kidney volume (cm^3^)	154.9 ± 7.4 (*N* = 25) (median = 161.0, range: 95.0–240.0)
Received LDKT	37.0% (17/46)
LD relation	
Father	23.5% (4/17)
Mother	47.1% (8/17)
Others	29.4% (5/17)
Donor age (y)	28.6 ± 1.6 (*N* = 46) (median = 26.5, range: 14.0–60.0)
Right donor kidney graft	43.5% (20/46)
Kidney graft single artery	80.4% (37/46)
Kidney graft single vein	97.8% (45/46)
Kidney biopsy: % of GS	3.1 ± 1.1 (*N* = 34) (median = 0.0, range: 0.0–26.0)
Perioperative/surgical technique	
L/NK mobilized	56.5% (26/46)
Surgical intervention pretransplant	56.5% (26/46)
Bladder augmentation	13.0% (6/46)
Arterial anastomosis with CIA	58.7% (27/46)
Venous anastomosis with IVC	56.5% (26/46)
WIT (min)	30.5 ± 1.0 (*N* = 46) (median = 29.5, range: 17.0–48.0)
CIT (h)	15.0 ± 1.8 (*N* = 46) (median = 19.3, range: 0.6–35.9)
EBL (cc)	25.1 ± 1.1 (*N* = 46) (median = 20.0, range: 10.0–200.0)

*RRT* renal replacement therapy, *ESRD* end-stage renal disease, *LDKT* living donor kidney transplant, *GS* glomerulosclerosis, *CIA* common iliac artery, *IVC* inferior vena cava, *WIT* warm ischemia time, *CIT* cold ischemia time, *EBL* estimated blood loss

The renal allograft length mean was 11.1 ± 0.2 cm (median 11.0, range: 9.0–14.0 cm), with a mean volume of 154.9 ± 7.4 cm^3^ (median 161.0, range: 95.0–240.0). The percentage of recipients that received a kidney from living donors was 37.0% (17/46), mostly from the mother (47.1%, 8/17). The mean donor age was 28.6 ± 1.6 years (median 26.5, range: 14.0–60.0 years). The right kidney was used for transplant in 43.5% of cases (20/46). A single artery and a single vein were encountered in 80.4% (37/46) and 97.8% (45/46) of cases, respectively. The mean percentage of glomerulosclerosis identified on the allograft explant biopsy was 3.1 ± 1.1% (median 0.0%, range: 0.0–26.0).

Regarding perioperative data, the L/NK mobilization technique was utilized in 56.5% (26/46) of patients (Group 1); the remaining 44.5% (20/46) of patients belonged to Group 2. Of the 46 pediatric patients, 26 (56.5%) had a history of surgical intervention, and 13.0% (6/46) had bladder augmentation prior to transplantation. The common iliac artery was used for arterial anastomosis in 58.7% (27/46) of cases, and the IVC was used in 56.5% (26/46) of the venous anastomoses. The mean warm ischemia time was 30.5 ± 1.0 min (median 29.5, range: 17.0–48.0), and the mean cold ischemia time was 15.0 ± 1.8 hours (median 19.3, range: 0.6–35.9 hours). The geometric mean estimated blood loss was 25.1 ± 1.1 cc (median 20.0, range: 10.0–200.0 cc).

The combined outcomes are outlined in Table 2. Only one (2.2%, 1/46) urological complication, Clavien > 3, was encountered in a patient (in Group 2) who developed a stricture at the ureterovesical junction at two weeks post-transplant, likely secondary to distal ureter ischemia, requiring a percutaneous radiological procedure with balloon ureteroplasty and nephroureteral stent placement. This patient eventually underwent surgical ureteral reimplantation at approximately 7 months post-transplant. No vascular or other complications were encountered during the study period. There were no cases of DGF or PNF. Among the 46 pediatric patients, 7 (15.2%, 7/46) developed biopsy-proven acute rejection (BPAR). The geometric mean serum Cr at 6 months post-transplant was 0.68 mg/dL ± 1.08 (median 0.69, range 0.21–2.40 mg/dL), and at 12 months post-transplant, it was 0.77 ± 1.08 mg/dL (median 0.72, range: 0.28–5.90 mg/dL), with a mean eGFR of 105.4 ± 4.3 mL/min/1.73 m^2^ (median 105.4, range 11.4–170.9 mL/min/0.73 m^2^). Two (4.3%) (death-censored) graft failures were observed, and there was one observed death (2.2%) with a functioning graft. The median follow-up among 43 patients who were alive with a functioning graft at the last follow-up was 24.6 (range 3.4–72.7) months post-transplant.

**Table 2:** Distributions of selected of clinical outcome variables (*N* = 46)

Baseline variables	Percentage with characteristic for categorical variables mean ± SE for (geometric mean ± SE for skewed) continuous variables; (median and range are included for continuous variables)
Development of a post-operative complication (Clavien Grade ≥ 3)	2.2% (1/46)
Developed DGF	0.0% (0/46)
Experienced PNF	0.0% (0/46)
Developed an acute rejection	15.2% (7/46)
Serum Cr at 6 mon (mg/dL)^a^	0.68 ± 1.08 (*N* = 43) (median = 0.69, range: 0.21–2.40)
Serum Cr at 12 mon (mg/dL)^a^	0.77 ± 1.08 (*N* = 41) (median = 0.72, range: 0.28–5.90)
eGFR at 12 mon(mL/min/1.73 m^2^)^a,b^	105.4 ± 4.3 (*N* = 41) (median = 105.4, range: 11.4–170.9)
(Death-censored) graft failure^c^	4.3% (2/46)
Death with a functioning graft^c^	2.2% (1/46)
(Death-uncensored) graft loss^c^	6.5% (3/46)

*DGF* delayed graft function, *PNF* primary nonfunction

^a^Patients who developed graft failure (i.e., return to permanent dialysis) prior to the time point analyzed for serum Cr were not included in the calculation

^b^eGFR at 12 mon was calculated using Schwartz’s original formula (= k*height at 12 months/serum Cr at 12 mon, where *k* = 0.55 if age < 13 y or female, 0.70 if age ≥ 13 y and male)

^c^Among 43 patients who were alive with a functioning graft at last follow-up, median follow-up was 24.6 (range: 3.4–72.7) mon post-transplant

### Management of rejection

During the study period, there were seven cases of rejection that were diagnosed based on allograft biopsy. Management varied based on the type of rejection and the discussion between the multidisciplinary team. Two patients from Group 1 who developed acute T-cell mediated rejection (1A) were treated with pulse steroids (methylprednisolone IV 10 mg/kg/day for two doses, followed by 5 mg/kg/day and a steroid taper). Another patient from Group 1 developed acute and chronic T-cell mediated rejection (1B) and was treated with pulse steroids as above and antithymocyte globulin (1 mg/kg/day for one day followed by 0.5 mg/kg/day for a second day). A fourth patient with rejection in Group 1 had acute T-cell mediated rejection (1B) with C4d positive staining concerning antibody-mediated rejection; he received pulse steroids, rituximab 325 mg IV once, two doses of IV immunoglobulin 500 mg/kg/day followed by four sessions of total plasma exchange (TPE). In the Group 2, one patient developed acute and chronic T-cell mediated rejection (1A), which was suspected to be related to overt immunosuppression nonadherence based on low serum tacrolimus trough levels and as described by the patient’s family. This patient received pulse steroids with methylprednisolone 500 mg IV/daily for three days, followed by antithymocyte globulin (2 mg/kg/day for 3 days) and one dose of rituximab 600 mg IV. Unfortunately, this graft did not respond and the patient returned to permanent dialysis (i.e., had graft failure) at 4.6 years post-transplant. A second patient was diagnosed with acute and chronic T-cell-mediated (1B) and antibody-mediated rejection; this patient was treated with pulse steroids (methylprednisolone 10 mg/kg/day followed by 5 mg/kg/day and a taper), IV immunoglobulin 500 mg/kg/day for two days and 400 mg/kg/day for a third day, and one dose of rituximab 100 mg IV. This graft also did not recover and had graft failure at 13.3 months post-transplant. A third patient with rejection in Group 2 developed acute T-cell mediated rejection (1B) and had positive donor specific antibodies (DSA); this patient was treated with pulse steroids (methylprednisolone 500 mg/day IV for 2 days, followed by 250 mg IV once and a steroid taper, antithymocyte globulin 1 mg/kg IV once, and rituximab 600 mg IV once).

No routine follow-up biopsies were performed. The patients were followed clinically, and improvement of renal function was used as objective data for improvement of the rejection episode.

### Group analysis

Comparisons of baseline recipient characteristics between the two groups are shown in Table 3. Patients in Group 1 were significantly younger than those in Group 2: 6.7 ± 0.8 vs. 15.3 ± 0.7 years (*P* < 0.001), had a shorter stature of 109.5 ± 3.7 vs. 154.2 ± 3.8 cm (*P* < 0.001), and weighed less, 21.4 ± 2.0 vs. 48.6 ± 3.4 kg (*P* < 0.001). The majority of the patients in Group 2 (95.0%, 19/20) were on dialysis prior to the transplant in comparison with Group 1 (69.2%, 18/26, *P* = 0.03), without a statistically significant difference between the years on RRT between the two groups (2.0 ± 0.4 vs. 1.7 ± 0.3 years, *P* = 0.60). None of the other recipient characteristics appeared to differ by group.

**Table 3:** Associations of baseline recipient variables with L/NK mobilization status. *RRT* renal replacement therapy, *ESRD* end-stage renal disease, *L/NK* liver/native kidney, *SE* standard error

Baseline variables	Percentage with characteristic for categorical variables (mean ± SE for continuous variables and geometric mean ± SE for skewed)		
	With L/NK mobilization (*N* = 26)	Without L/NK mobilization (*N* = 20)	*P* value
Recipient male	65.4% (17/26)	50.0% (10/20)	0.29
Recipient age (y)	6.7 ± 0.8 (*N* = 26)	15.3 ± 0.7 (*N* = 20)	< 0.001
Received RRT	69.2% (18/26)	95.0% (19/20)	0.03
Years on RRT (0 if none)	1.7 ± 0.3 (*N* = 26)	2.0 ± 0.4 (*N* = 20)	0.60
Recipient height (cc)	109.5 ± 3.7 (*N* = 26)	154.2 ± 3.8 (*N* = 20)	< 0.001
Recipient weight (kg)	21.4 ± 2.0 (*N* = 26)	48.6 ± 3.4 (*N* = 20)	< 0.001
ESRD diagnosis			0.03
Pre-renal	3.8% (1/26)	5.0% (1/20)	
Renal	38.5% (10/26)	65.0% (13/20)	
Post-renal	26.9% (7/26)	5.0% (1/20)	
Combined	30.8% (8/26)	10.0% (2/20)	
Unknown	0.0% (0/26)	15.0% (3/20)	
Recurrent ESRD	0.0% (0/26)	10.0% (2/20)	0.10

Comparisons of donor characteristics are shown in Table 4. Of the 26 patients in Group 1, 10 (38.5%) received an LD kidney vs. 35.0% (7/20) of Group 2 patients who received an LD kidney (*P* = 0.81). The median donor ages for Groups 1 and 2 were 27.6 ± 2.2 years and 30.0 ± 2.4 years, respectively (*P* = 0.48). The right kidney was used in 38.5% (10/26) of cases in Group 1 vs. 50.0% (10/20) in Group 2 (*P* = 0.43). None of the other donor characteristics appeared to differ by group.

**Table 4:** Associations of baseline donor variables with liver/native kidney (L/NK) mobilization status *ESRD* end-stage renal disease, *LDKT* living donor kidney transplant, *GS* glomerulosclerosis, *SE* standard error

Baseline variables	Percentage with characteristic for categorical variables (mean ± SE for geometric mean ± SE for skewed continuous variables)		
	With L/NK mobilization (*N* = 26)	Without L/NK mobilization (*N* = 20)	*P* value
Kidney length (cm)	11.2 ± 0.3 (*N* = 23)	11.0 ± 0.3 (*N* = 14)	0.78
Kidney volume (cm^3^)	153.2 ± 10.9 (*N* = 13)	156.7 ± 10.4 (*N* = 12)	0.82
Received LDKT	38.5% (10/26)	35.0% (7/20)	0.81
LD relation			0.15
Father	40.0% (4/10)	0.0% (0/7)	
Mother	40.0% (4/10)	57.1% (4/7)	
Others	20.0% (2/10)	42.9% (3/7)	
Donor age (y)	27.6 ± 2.2 (*N* = 26)	30.0 ± 2.4 (*N* = 20)	0.48
Right donor kidney draft	38.5% (10/26)	50.0% (10/20)	0.43
Kidney graft single artery	84.6% (22/26)	75.0% (15/20)	0.42
Kidney graft single vein	100.0% (26/26)	95.0% (19/20)	0.25
Kidney biopsy: % of GS	3.8 ± 1.6 (*N* = 22)	1.8 ± 0.8 (*N* = 12)	0.28

The perioperative and surgical technique data comparisons are shown in Table 5. As expected, there was a statistically significant difference when comparing the history of bladder augmentation between Group 1 (23.1%, 6/26) and Group 2 (0.0%. 0/20, *P* = 0.02). In addition, the majority of the arterial anastomosis in Group 1 (92.3%, 24/46) was performed with CIA vs. only 15.0% (3/20) of the time in Group 2 (*P* < 0.001). The IVC was used in 100% (26/26) of patients as a target for venous anastomosis in Group 1 vs. 0.0% (0/20) in Group 2 (*P* < 0.001). None of the other differences, including those for warm ischemia time, cold ischemia time, and estimated blood loss, were statistically significant between groups (*P* = 0.80, 0.84 and 0.29, respectively).

**Table 5:** Associations of perioperative and surgical technique variables with L/NK mobilization status *CIA* common iliac artery, *IVC* inferior vena cava, *WIT *warm ischemia time, *CIT* cold ischemia time, *EBL* estimated blood loss, *L/NK* liver/native kidney

Baseline variables	Percentage with characteristic for categorical variables (mean ± SE for geometric mean ± SE or skewed continuous variables)		
	With L/NK mobilization (*N* = 26)	Without L/NK mobilization (*N* = 20)	*P* value
Surgical intervention pretransplant	65.4% (17/26)	45.0% (9/20)	0.17
Bladder augmentation	23.1% (6/26)	0.0% (0/20)	0.02
Arterial anastomosis with CIA	92.3% (24/26)	15.0% (3/20)	< 0.001
Venous anastomosis with IVC	100.0% (26/26)	0.0% (0/20)	< 0.001
WIT (min)	30.3 ± 1.0 (*N* = 26)	30.8 ± 1.9 (*N* = 20)	0.80
CIT (h)	14.7 ± 2.3 (*N* = 26)	15.4 ± 2.8 (*N* = 20)	0.84
EBL (cc)	22.8 ± 1.1 (*N* = 26)	28.4 ± 1.2 (*N* = 20)	0.29

As described above, only one post-operative complication was encountered, a patient from Group 2 who developed a (Clavien Grade 3b) ureteral stricture requiring surgical repair; no other urologic, vascular, or surgical complication was found, and there was no statistically significant difference between the two groups regarding post-operative complications (*P* = 0.25). No patient developed DGF or experienced PNF during the study period. Details of the clinical outcome comparisons are shown in Table 6.

**Table 6:** Associations of clinical outcomes with liver/native kidney (L/NK) mobilization status

Outcome variables	Percentage with characteristic for categorical variables (mean ± SE for continuous variables geometric mean ± SE or skewed)		
	With L/NK mobilization (*N* = 26)	Without L/NK mobilization (*N* = 20)	*P* value
Development of a post-operative complication (Clavien Grade ≥ 3)	0.0% (0/26)	5.0% (1/20)	0.25
Developed DGF	0.0% (0/26)	0.0% (0/20)	1.00
Experienced PNF	0.0% (0/26)	0.0% (0/20)	1.00
Developed an acute rejection	15.4% (4/26)	15.0% (3/20)	0.79^c^
Serum Cr at 6 mon (mg/dL)^a^	0.5 ± 1.1 (*N* = 24)	1.0 ± 1.1 (*N* = 19)	< 0.001
Serum Cr at 12 mon (mg/dL)^a^	0.6 ± 1.1 (*N* = 23)	1.1 ± 1.1 (*N* = 18)	0.0003
eGFR at 12 mon (mL/min/1.73 m^2^)^a,b^	111.0 ± 4.9 (*N* = 23)	98.3 ± 7.5 (*N* = 18)	0.15
Death-censored graft failure	0.0% (0/26)	10.0% (2/20)	0.22^c^
Death with a functioning graft	0.0% (0/26)	5.0% (1/20)	0.21^c^
Death-uncensored graft loss	0.0% (0/26)	15.0% (3/20)	0.09^c^

*DGF* delayed graft function, *PNF* primary nonfunction, *eGFR* glomerular filtration rate

^a^Patients who developed graft failure (i.e., return to permanent dialysis) prior to the time point analyzed for serum Cr were not included in the calculation

^b^eGFR at 12 mon was calculated using Schwartz’s original formula (= *k* × height at 12 mon/Serum Cr at 12 mon, where *k* = 0.55 if age < 13 y or female, 0.70 if age ≥ 13 y and male)

^c^Log-rank test *P* value

Regarding BPAR occurrence, 15.4% (4/26) of patients in the Group 1 were diagnosed and treated vs. 15.0% (3/20) in the Group 2 (*P* = 0.79). The geometric mean serum creatinine at 6 months post-transplant for the two groups was 0.5 ± 1.1 (*n* = 24) and 1.0 ± 1.1 (*n* = 19), which was statistically significant (*P* < 0.001). Similar results were found when comparing serum creatinine at 12 months post-transplant: 0.6 ± 1.1 (*n* = 23) vs. 1.1 ± 1.1 (*n* = 18, *P* = 0.0003). However, when controlling serum Cr for patient size (i.e., height, age, and sex) using Schwartz’s original formula, the mean eGFR at 12 months post-transplant was not significantly different between the 2 groups: 111.0 ± 4.9 mL/min/1.73 m^2^ (*n* = 23) in Group 1 vs. 98.3 ± 7.5 mL/min/1.73 m^2^ (*n* = 18) in Group 2 (*P* = 0.15).

There were two cases (10.0%, 2/20) of death-censored graft failure in the Group 2 vs. none (0.0%, 0/26) in the Group 1. As described above, their causes were related to severe rejection. One patient developed rejection following overt immunosuppression nonadherence and returned to permanent hemodialysis at 4.6 years post-transplant. The other patient developed acute T-cell and antibody-mediated rejection that progressed to chronic kidney disease; this patient was placed back on permanent hemodialysis at 13.3 months post-transplant. One death with a functioning graft due to a cardiovascular event occurred for one patient in Group 2 at 15.6 months post-transplant. There were no statistically significant differences when comparing the rates of death-censored graft failure or death with a functioning graft between the two groups (*P* = 0.22 and 0.21, respectively).

No complications, such as ileus, bowel obstruction, wound complications (superficial/deep), perirenal collection, lymphocele, hematoma, subcutaneous seroma, or wound dehiscence, were observed.

Regarding maintenance immunosuppression, tacrolimus was initially used in 100% of the patients. Steroid-free immunosuppression was used in 38/46 (83%) patients soon after transplant. Mycophenolate mofetil was initiated in 45/46 (98%) of patients but was changed to azathioprine (*n* = 3), sirolimus (*n* = 3), tacrolimus monotherapy (*n* = 1) and prednisone (*n* = 2) for intolerance, viral infection or leukopenia. Prednisone was also given for maintenance in patients with a primary diagnosis of vasculitis (*n* = 5) and a high degree of sensitization (*n* = 1) and was subsequently added for maintenance therapy in patients (*n* = 7) who rejected.

## Discussion

**Fig. 3:**
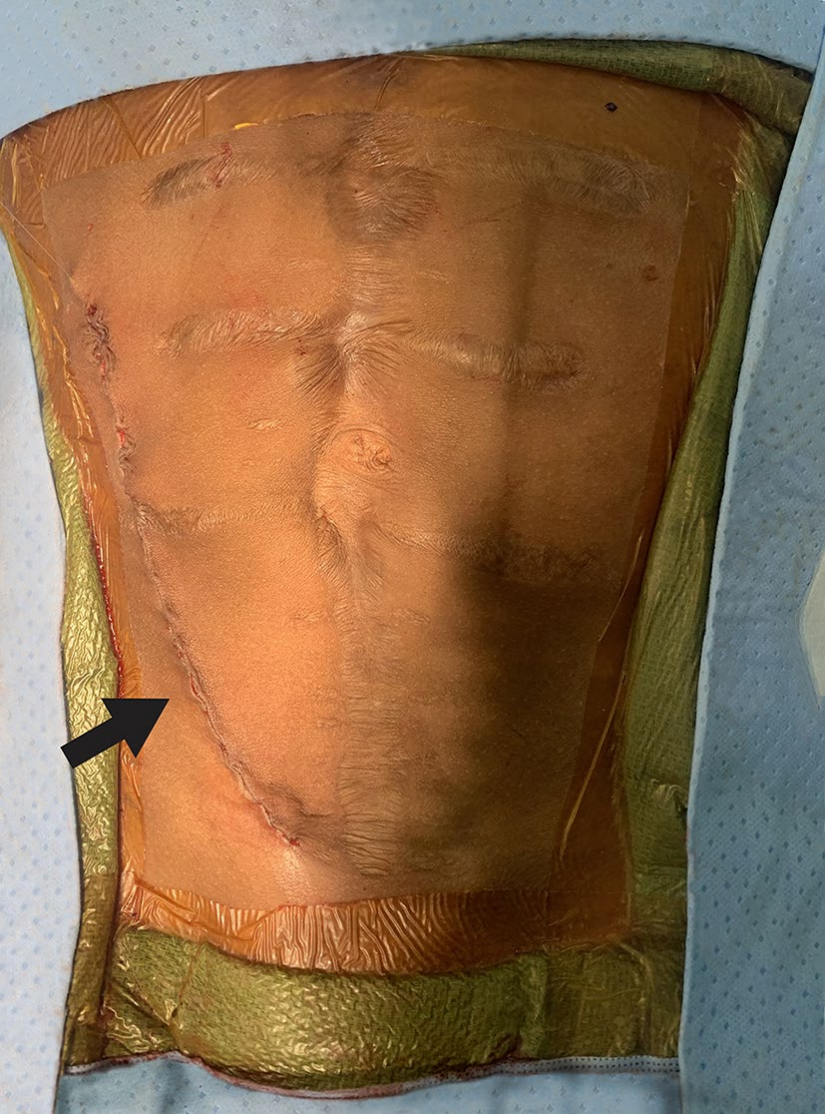
Gibson incision with retroperitoneal placement of the deceased donor kidney. The recipient underwent multiple abdominal surgeries

A retroperitoneal approach for pediatric KT is well established and has been shown to lower the risk of abdominal compartment syndrome and non-vascular complications when compared to an intraperitoneal approach [8, 20]. In a retrospective review by Taher et al. [20], 146 pediatric patients underwent kidney transplantation, 57 (39%) with an intraperitoneal approach were found to have an overall intraabdominal complication rate of 29 (17/57)%. Twelve patients of 146 (8.2%) developed gastrointestinal complications, ranging from bowel obstruction to volvulus. The risk factors identified were intraperitoneal transplant surgical technique and history of previous abdominal operations (Fig. 3), which is relatively common in this patient population.

The retroperitoneal technique has been previously described in detail by Alameddine et al. [11], where the native kidney and liver are mobilized to accommodate sufficient space for the recipient to receive the adult allograft. This technique also avoids native kidney nephrectomy, which is associated with higher blood transfusion requirements, prolonged hospitalization, and intraoperative complications [21]. Previous reports [22, 23] have shown that intraabdominal placement of the renal allograft increases the risk of kidney torsion with subsequent graft loss. Hence, the retroperitoneal space protects the kidney from twisting, while the associated scarring and fibrosis between the graft and retroperitoneal raw surface keeps it in place [23].

Gargah et al. [24] analyzed 82 consecutive pediatric retroperitoneal kidney transplants and obtained an incidence of vascular complications of 8.5%, while other studies [25–27] determined rates ranging from 0.5 to 6.2%. In our study, as of the last follow-up date, we did not observe any vascular complications, thrombosis, or renal artery stenosis.

Another aspect of our surgical technique is stentless ureteral anastomosis. Our center, as described by Ciancio et al. [12], performs a modified extravesical ureteroneocystostomy technique with lower urologic complications and avoids the routine use of ureteral stent placement, minimizing the need to perform additional procedures and possibly also decreasing the risk of urinary tract infections. In this study, we encountered a ureteral stricture in one pediatric patient, likely secondary to distal ureteral ischemia, which was unlikely to be prevented by a ureteral stent. This suggests a complication rate of 2.2% (1/46), which appears to be favorable when compared with other published series [28, 29] that report a 5% to 13% incidence of urologic complications.

There were no surgical drains utilized in any of the patients in our study, and no complications were observed due to the absence of the drain. Careful and minimal dissection is necessary when exposing the vasculature to avoid unnecessary lymph leakage and fluid collection. There were no collections observed in any baseline or follow-up ultrasounds of the kidney allografts [14].

As expected, recipients from Group 1 had younger age and lower weight when compared to Group 2, because the smaller patients required both native kidney and liver mobilization to create an adequate size pocket for the kidney allograft. Despite the challenges imposed by children with a smaller size requiring further dissection, there was no statistically significant difference noted in the complication rate when comparing both groups. This also explains the significant difference between the groups when comparing serum creatinine levels, as Group 2 consisted of older and larger children, leading to greater muscle mass and higher serum levels of creatinine. When using Schwartz’s original formula to calculate the eGFR at 12 months, we did not encounter a statistically significant difference between the two groups [18, 30].

We did not encounter any case of DGF or graft PNF. The common allocation of low kidney donor profile index (KDPI) kidney grafts to the pediatric patient population, the utilization of grafts from living donation, and the routine use of pulsatile machine perfusion (Life-Port ^®^) when a DD renal allograft is retrieved [31, 32] all contribute to the lower incidence of DGF and graft PNF [33–35].

To our knowledge, there are no other studies that compared the outcomes of pediatric patients with and without L/NK mobilization. Muramatsu et al. [36] published a comparison between intraperitoneal vs. retroperitoneal placement of the adult kidney allograft in small pediatric patients. They did not encounter a statistically significant difference in 5-year graft survival between groups; however, the intraperitoneal group had an incidence of ileus in 8.3% of the patients, and a native kidney nephrectomy was required in almost 66% of the intraperitoneal group and even 48.1% in the retroperitoneal group at the time of transplant. Hence, L/NK mobilization might mitigate these issues.

Limitations of our study are primarily due to the retrospective nature, non-randomization and small sample size. Another limitation is that the study was performed by a single surgeon at a single center, which might limit generalizability; however, this approach eliminated differences in techniques and possibly made the outcomes (and group comparisons) less heterogeneous.

In summary, the use of L/NK mobilization is an acceptable and safe approach for pediatric KT recipients receiving adult allografts in the extraperitoneal space. Our study demonstrates that this maneuver facilitates placement of an adult allograft graft in pediatric patients with no increase of developing post-operative complications, graft loss, or mortality when compared to our extraperitoneal technique without L/NK mobilization.

## Data Availability

The raw data supporting the conclusion of this article will be made available by the authors, without undue reservation.
